# Supplementary material for: Bruce suppresses autophagy-regulated caspase activity and wing tissue growth in Drosophila
Source: eLife. 2026 Oct 19;15:RP109876. doi: 10.7554/eLife.109876 (PMC13679926; doi:10.7554/eLife.109876)
Supplement: Peer review history

# Editor report: eLife Assessment

**DOI:** 10.7554/eLife.109876.3.sa0

**Authors:** Erika A Bach

**Affiliations:** NYU Grossman School of Medicine United States

**Keywords:** Compelling, Important

This **important** study reports insights into how the caspase Dcp-1, best known for cell death, can also promote tissue growth in *Drosophila*, extending the authors' earlier work by identifying regulatory factors that shape this non-lethal activity. The **compelling** findings identify a physical and functional interaction between Dcp-1 and Bruce, as well as new Dcp-1-interacting proteins that function in autophagy: Sirt1, Fkbp59, Debcl, Buffy, Atg2, and Atg8a. This work helps broaden the understanding of the non-lethal roles of Dcp-1.

# Referee report: Reviewer #1 (Public review)

**DOI:** 10.7554/eLife.109876.3.sa1

The authors clearly demonstrate that overexpressed Dcp-1, but not Drice, is activated without canonical apoptosome components.

Using TurboID-based proximity labeling they revealed distinct proximal proteomes, among which Sirtuin 1, an Atg8a deacetylase, which promotes autophagy, was specifically required for Dcp-1 activation. Additionally, the show that autophagy-related genes, including Bcl-2 family members Debcl and Buffy, are required for Dcp-1 activation. Using structure-based prediction using AlphaFold3 they identified that Bruce, an autophagy-regulated inhibitor of apoptosis, as a Dcp-1-specific regulator acting outside the apoptosome-mediated pathway. Finally, they show that Bruce suppresses wing tissue growth. These findings indicate that non-lethal Dcp-1 activity is governed by the autophagy- Bruce axis, enabling distinct non-lethal functions independent of cell death.

Comments on revised version.

No further comments.

# Referee report: Reviewer #2 (Public review)

**DOI:** 10.7554/eLife.109876.3.sa2

Summary:

The *Drosophila* executioner caspase Dcp-1 has established roles in cell death, autophagy, and imaginal disc growth. This study reports previously unrecognized factors that work together with Dcp-1. Specifically, the authors performed a turboID-based proximal ligation experiment to identify factors associated Dcp-1 and Drice. Dcp-1-specific interactors were further examined for their genetic interaction. The authors report autophagy-related genes, including Debcl and Buffy, to be required for Dcp-1 activation. In addition, the authors present evidence of an interaction between Bruce and Dcp-1. Bruce expression blocks the Dcp-1 overexpression phenotype. Inhibition of effector caspases or overexpression of Bruce commonly reduced wing growth, suggesting a relationship between the two proteins.

Strengths:

The study identifies new Dcp-1-interacting proteins and provides a functional link between Dcp-1 and Sirt1, Fkbp59, Debcl, Buffy, Atg2, and Atg8a. During the revision, the authors have also added convincing new data supporting the interaction between Dcp-1 and Bruce. They further make a strong case regarding the quality of the turboID-proteomics data. Overall, this is a strong manuscript supporting an interesting discovery.

# Referee report: Reviewer #3 (Public review)

**DOI:** 10.7554/eLife.109876.3.sa3

Summary:

The present paper by Shinoda et al. from the Miura group builds upon findings reported in an earlier study by the same team (Shinoda et al., PNAS, 2019), which identified a non-apoptotic role for the *Drosophila* executioner caspase Dcp-1 in promoting wing tissue growth. That earlier work attributed this function primarily to Dcp-1 and to Decay, a caspase structurally related to executioner caspases, but not to DrICE, the principal apoptotic executioner caspase. The authors further proposed that this non-apoptotic caspase activity operates independently of the initiator caspase Dronc.

In the current study, the authors both corroborate aspects of their previous findings and extend the investigation to mechanisms regulating Dcp-1 in this context. They identify roles for the giant IAP Bruce, two BCL-2 family members, and autophagy-related components in modulating non-apoptotic Dcp-1 activity. Moreover, they show that Bruce binds to a BIR-like peptide exposed upon Dcp-1 cleavage, but not to DrICE. The study further suggests that low levels of Dcp-1 activity promote wing tissue growth, whereas excessive activity induces cell death, as evidenced by impaired wing development following Dcp-1 overexpression. Overall, the manuscript provides several intriguing insights into the non-apoptotic regulation of the comparatively weak apoptotic executioner caspase Dcp-1 and complements the group's earlier work. However, several concerns remain regarding certain interpretations of the data and the experimental rigour of some of the results.

Strengths:

A major strength of the work is its systematic genetic and biochemical approaches, which combine tissue-specific manipulation with protein interaction mapping to explore how Dcp-1 is regulated. The identification of several regulatory factors, including an inhibitor of cell death protein and components linked to autophagy, provides a coherent framework for understanding how Dcp-1 activity might be tuned.

Weaknesses:

The evidence supporting some key claims remains incomplete. In particular, the type of cell death form induced when Dcp-1 is overexpressed is not clearly established, and additional tests would be needed to distinguish between the different cell death types.

Likely impact:

The study contributes to a growing body of work showing that proteins traditionally associated with cell death can have broader roles in tissue development. This conceptual advance is likely to be of interest to researchers studying growth control and tissue maintenance.

Specific points:

(1) Nature of the wing ablation phenotype

A central concern is whether the wing ablation phenotype observed upon Dcp-1 overexpression truly reflects apoptotic cell death. The authors show in Fig. 1c that nuclei in cells overexpressing Dcp-1, but not DrICE, zymogens are highly condensed, which is suggestive of apoptosis. However, it is equally plausible that this phenotype reflects a form of non-apoptotic, Dcp-1-dependent cell death (e.g. autophagy-dependent cell death). This distinction could be readily addressed using TUNEL labelling and direct caspase activity assays. The latter would be particularly informative, as it remains unclear whether zymogen Dcp-1 is capable of cleaving standard effector caspase reporters in vivo. Does the anti-cleaved Dcp-1 antibody detect Dcp-1 activation following overexpression of the Dcp-1 zymogen?

(2) Role of Decay

In their earlier study, the authors identified Decay as another caspase influencing wing growth, albeit more modestly than Dcp-1. It is therefore unclear why this line of investigation was not pursued further in the current work. This omission is notable, as Decay is not implicated in apoptosis and, to date, no substantial physiological function has been assigned to this caspase in any system. At minimum, this point should be discussed explicitly.

(3) Fig. 2: Proximity labelling analysis

The authors use TurboID-mediated proximity labelling to reveal distinct Dcp-1- and DrICE-associated proteomes across tissues, with a particular focus on the wing disc. They further demonstrate that RNAi-mediated knockdown of the Dcp-1-associated proteins Sirt1 and Fkbp59 suppresses the wing ablation phenotype induced by Dcp-1 overexpression, suggesting that these factors are required for Dcp-1 activity. However, it should be clarified whether Bruce was identified as a Dcp-1 interactor in the proximity labelling dataset, given its proposed central regulatory role. In addition, further discussion of Fkbp59, its known functions and how it might mechanistically influence Dcp-1 activity, would be valuable.

(4) Fig. 3: Autophagy-related factors

Given that Sirt1 is known to promote autophagy, the authors next examine autophagy-related proteins and identify roles for Atg2, Atg8a, Debcl, and Buffy in Dcp-1 activation. Notably, these proteins do not promote cell death in the Hid-induced canonical apoptotic pathway. However, it is important to determine whether knockdown of Debcl, Buffy, Atg2, or Atg8a alone affects wing development in the absence of Dcp-1 overexpression, to exclude the possibility that these perturbations independently impair wing formation.

(5) Evidence for canonical autophagy

The involvement of autophagy would be more convincingly demonstrated by testing additional core autophagy genes, such as Atg7, Atg5, and Atg12, as well as performing a combined knockdown of Atg8a and Atg8b. Moreover, direct assessment of autophagy at the cellular level using established genetic reporters would substantially strengthen the conclusions.

(6) Figs. 4-5: Functional consequences

It would be informative to determine whether Synr, Debcl, or Buffy influence wing size on their own and whether their overexpression enhances wing growth.

(7) Terminology and interpretation of cell death

Taken together, the results suggest that Dcp-1 zymogen overexpression induces a form of non-apoptotic cell death, potentially autophagy-dependent or related. The reviewer does not understand the authors' insistence on referring to this process as apoptosis. The authors should be more cautious in their terminology: there is no canonical versus non-canonical apoptosis, there is simply apoptosis. Without stronger evidence, these effects should not be described as apoptotic cell death.

Comments on revised version.

In the revised manuscript, the authors addressed each of my concerns in good faith and, in my opinion, responded to them thoroughly and satisfactorily. I have no further concerns.

# Author comment: Author response

**DOI:** 10.7554/eLife.109876.3.sa4

**Authors:** Natsuki Shinoda, Yutaro Hama, Nozomi Hanawa, Masayuki Miura

**Affiliations:** National Institute of Advanced Industrial Science and Technology Ibaraki Japan; University of Tsukuba Ibaraki Japan; The University of Tokyo Tokyo Japan; The University of Tokyo Tokyo Japan

The following is the authors’ response to the original reviews.

We are grateful to all the reviewers for dedicating time to review our manuscript and for providing insightful comments and suggestions. We have revised our manuscript in line with the reviewers' feedback. The major revisions include characterization of Dcp-1 overexpression-induced cell death, demonstration of the involvement of autophagy in Dcp-1 activation, characterization of the interaction between full-length Bruce and cleaved Dcp-1. We have introduced new figures (Figure 1 – figure supplement 1, Figure 2 – figure supplement 2, Figure 3 – figure supplement 1, Figure 5 – figure supplement 1), new panels (Figures 1D, Figure 3C, Figure 4I, J) and a new table (Table S2). The previous Figure 5 – figure supplement 1 has been relocated to Figure 4 – figure supplement 2.

With all concerns and suggestions from the reviewers addressed, our conclusion—that Bruce suppresses autophagy-regulated caspase activity and wing tissue growth in *Drosophila*— is now more robustly supported. We are confident that our revised manuscript makes a significant contribution to the fields of cell death, autophagy, and developmental biology, as it provides a new conceptual framework for understanding non-lethal caspase regulation. We remain hopeful that the reviewers will find it suitable for publication in eLife.

**Reviewer #1 (Public review):**

Summary:

The authors clearly demonstrate that overexpressed Dcp-1, but not Drice, is activated without canonical apoptosome components. Using TurboID-based proximity labeling, they revealed distinct proximal proteomes, among which Sirtuin 1, an Atg8a deacetylase, which promotes autophagy, was specifically required for Dcp-1 activation. Additionally, the show that autophagy-related genes, including Bcl-2 family members Debcl and Buffy, are required for Dcp1 activation. Using structure-based prediction using AlphaFold3, they identified that Bruce, an autophagy-regulated inhibitor of apoptosis, acts as a Dcp-1-specific regulator acting outside the apoptosome-mediated pathway. Finally, they show that Bruce suppresses wing tissue growth. These findings indicate that non-lethal Dcp-1 activity is governed by the autophagy-Bruce axis, enabling distinct non-lethal functions independent of cell death.

Strengths:

This is an excellent paper with very good structure, excellent quality data and analysis.

Weaknesses:

This reviewer did not identify any weaknesses or recommendations for revision.

We sincerely thank the reviewer for their highly positive evaluation of our work. We are pleased that the reviewer found the overall structure, data quality, and analyses to be strong, and that they clearly recognized the key findings of our study. No changes to the manuscript were required in response to this review.

**Reviewer #2 (Public review):**

Summary:

The *Drosophila* executioner caspase Dcp-1 has established roles in cell death, autophagy, and imaginal disc growth. This study reports previously unrecognized factors that work together with Dcp-1. Specifically, the authors performed a turboID-based proximal ligation experiment to identify factors associated Dcp-1 and Drice. Dcp-1-specific interactors were further examined for their genetic interaction. The authors report autophagy-related genes, including Debcl and Buffy, to be required for Dcp-1 activation. In addition, the authors present evidence of an interaction between Bruce and Dcp-1. Bruce-expression blocks the Dcp-1 overexpression phenotype. Inhibition of effector caspases or overexpression of Bruce commonly reduced wing growth, suggesting a relationship between the two proteins.

Strengths:

On the positive side, the study identifies new Dcp-1-interacting proteins and provides a functional link between Dcp-1 and Sirt1, Fkbp59, Debcl, Buffy, Atg2, and Atg8a.

Weaknesses:

The data supporting the Dcp-1/Bruce interaction are not strong, even though the title of this manuscript highlights Bruce. For example, the authors' turboID data does not support Dcp1/Bruce interaction. The case for the interaction is based on a single experiment that overexpresses a truncated Bruce transgene in S2 cells.

We sincerely thank the reviewer for their constructive and detailed evaluation of our manuscript. We appreciate the positive assessment that our study identifies new Dcp-1-associated factors and provides functional links between Dcp-1 and Sirt1, Fkbp59, and multiple autophagy-related genes, including Debcl, Buffy, Atg2, and Atg8a. We also thank the reviewer for clearly pointing out concerns regarding the strength and interpretation of the evidence connecting Bruce and Dcp-1. In the revised manuscript, we have addressed these concerns in two major ways. First, we provided additional experimental evidence explaining why TurboID-mediated labeling did not identify Bruce. Specifically, we showed that the majority of TurboID-tagged Dcp-1 expressed in wing imaginal discs remains in its full-length form, which is unlikely to engage Bruce. Second, and more importantly, we now demonstrated that endogenously expressed full-length Bruce interacts with cleaved Dcp-1 in wing imaginal discs. These new data provide strong support for a physiologically relevant interaction between Bruce and cleaved Dcp-1. Detailed descriptions of these experiments and results are provided in the point-by-point responses in the “recommendations for the authors” section. Together, these newly added data substantially strengthen the evidence for the Dcp-1/Bruce interaction and support the focus of the original manuscript title.

**Reviewer #2 (Recommendations for the authors):**

(1) The title of the manuscript highlights Dcp-1/Bruce interaction, even though the evidence there is not strong. The evidence for Dcp-1/Sirt1 and Dcp-1/Fkbp59 is stronger. How about changing the title to highlight these other Dcp-1 interactions?

We thank the reviewer for the thoughtful suggestion. We agree that several Dcp-1-associated factors identified in our study, particularly Sirt1 and Fkbp59, are supported by functional evidence. Specifically, our data show that Sirt1 and Fkbp59 are required for *Dcp-1* overexpression-mediated activation. However, Bruce differs from these factors in both the scope and the nature of its effects on Dcp-1. Bruce is not only shown to specifically suppress Dcp-1 activity, but also to suppress wing tissue growth, indicating a broader physiological role in modulating non-lethal Dcp-1 function. Importantly, we further demonstrate that Bruce can specifically physically interact with cleaved Dcp-1. In addition, in this revised manuscript, we show that using the endogenously mStayGold::V5-tag knock-in-tagged Bruce allele, cleaved Dcp-1, induced by overexpression of Dcp-1::VENUS in wing imaginal discs, can be co-immunoprecipitated with full-length Bruce (new Figure 4I, J). These results support a physical interaction between full-length Bruce and activated Dcp-1 in vivo, consistent with a direct inhibitory role. Based on these findings, we decided to retain Bruce in the manuscript title, as it is the only factor for which both physiological and functional interactions with Dcp-1 are supported by multiple independent lines of evidence.

(2) The case for Dcp-1/Bruce interaction is not strong because the Dcp-1 turboID fails to identify Bruce. In fact, the Dcp-1 turboID approach may not have been effective, as it failed to detect many established interactions, including Diap1 (Wang et al. 1999 PMID 10481910; Tenev et al., 2006 PMID 15580265). The authors may want to comment on this.

We thank the reviewer for raising this important point. We agree that Bruce, as well as DIAP-1, was not identified in our TurboID-MS labeling dataset (Figure 2C, Table S1). Previous studies have shown that DIAP1 interacts with Dcp-1 and Drice only after exposure of the IAP-binding motif (IBM) at the neo-N-terminus of the large executioner caspase subunit following cleavage (Tenev et al., 2005). Similarly, our co-immunoprecipitation analyses show that Bruce interacts specifically with cleaved Dcp-1, but not with full-length Dcp-1. In the revised manuscript, we confirmed by western blot that the majority of endogenously expressed Dcp-1 in wing imaginal discs is present in the full-length pro-form (new Figure 2 – figure supplement 2A). Thus, the failure to identify Bruce and DIAP1 by TurboID-MS using full-length Dcp-1 as bait is expected, as this approach primarily labels interactors of the inactive, full-length form of Dcp-1. To evaluate whether our proximity labeling approach was nevertheless effective, we compared our TurboIDMS dataset with a previously published immune-affinity purification (IAP)-MS dataset generated using catalytically inactive, C-terminally V5-tagged Dcp-1 overexpressed in *Drosophila* 1(2)mbn cells (Choutka et al., 2017). Although the experimental conditions differ in several respects, we observed a substantial overlap between the TurboID-MS-mediated and IAP-MS-mediated interaction lists (new Figure 2 – figure supplement 2B, new Table S2). Importantly, SesB, one of the best-characterized Dcp-1 interactors located in mitochondria (DeVorkin et al., 2014), was also identified in our mass spectrometry dataset (new Figure 2 – figure supplement 2B, new Table S2). Based on these analyses, we now more explicitly describe the experimental context and limitations of the TurboID approach, clarifying that it preferentially labels interactors of full-length Dcp-1 in the revised manuscript. We also incorporate comparisons with prior studies to further support the validity of our mass spectrometry experiments in the revised manuscript

(3) The best experimental evidence for Bruce/Dcp-1 interaction can be found in Figure 4H. But here, they see a weak interaction only when a truncated Bruce construct is overexpressed in S2 cells. Whether Dcp-1 interacts with Bruce in a physiological setting remains unsupported.

We thank the reviewer for the important comment. We agree that, in the original manuscript, the biochemical evidence for the Bruce/Dcp-1 interaction relied primarily on experiments using an overexpressed truncated Bruce construct in S2 cells and therefore did not sufficiently establish whether this interaction occurs in vivo, especially in wing imaginal discs. To address this concern, we performed additional experiments to examine the Bruce/Dcp-1 interaction. In the background of the mStayGold::V5-tag knocked-in *Bruce* allele, we overexpressed Dcp-1::VENUS using *WPGal4* driver to induce Dcp-1 activation and tested whether full-length Bruce under endogenous expression interacts with cleaved Dcp-1 in wing imaginal discs. Following immunoprecipitation with anti-V5 antibody-conjugated magnetic agarose, we found that cleaved Dcp-1 signal was enriched by co-immunoprecipitation (new Figure 4I, J). These new data demonstrate that Bruce associates with cleaved Dcp-1 in vivo and thus support the physiological relevance of the Bruce/Dcp-1 interaction. We have clarified this point in the revised manuscript and included the corresponding data.

(4) The genetic interaction between Bruce and Dcp-1 is interesting, but the interpretation becomes complicated because Bruce inhibits Reaper, and at the same time, Dcp-1 genetically interacts with Reaper, Hid, and Grim (Figures 1E, F, G). Thus, it remains unclear if the genetic interaction between Bruce/Dcp-1 is due to a direct interaction between Bruce/Dcp-1 or alternatively, because Bruce inhibits Reaper and Grim.

We thank the reviewer for the comment. The primary function of Reaper, Hid, and Grim (RHG proteins), collectively referred to as IAP antagonists, is to directly interact with inhibitor of apoptosis proteins (IAPs), most notably DIAP-1 (Kornbluth and White, 2005; Ryoo and Baehrecke, 2010), leading to the inhibition of DIAP-1 function. RHG proteins have not been shown to directly inhibit caspases. Because inhibition of RHG proteins results in the stabilization of DIAP-1, it is likely that the effects observed upon *RHG* gene knockdown are mediated through DIAP-1. Consistent with this idea, overexpression of *DIAP-1*, while less potent than Bruce, can also suppress Dcp-1 activation (Figure 5B, C). However, we also acknowledge that Bruce suppresses Reaper- and Grim-dependent, but not Hid-dependent, cell death (Vernooy et al., 2002). In addition, Bruce directly targets Reaper through non-lysine ubiquitination, promoting its degradation (Domingues and Ryoo, 2012). Thus, it is possible that *Bruce* overexpression suppresses Reaper and thereby strengthens DIAP-1 function, which could indirectly contribute to the inhibition of Dcp-1 activation. Nevertheless, because the effect of Bruce overexpression is stronger than that of DIAP-1 overexpression (Figure 5B, C), and together with our physical interaction data of Bruce with cleaved Dcp-1, we propose that Bruce most likely inhibits Dcp-1 directly to attenuate its activation.

(5) In general, the manuscript could benefit from highlighting the strong data on Sirt1 and Fkbp59, while clearly acknowledging the limitations of the Bruce/Dcp-1 interaction.

We thank the reviewer for the comment. As described above, in the revised manuscript we now demonstrate that endogenously expressed full-length Bruce physically interacts with cleaved Dcp-1 in wing imaginal discs (Figure 4I, J). These new data provide strong support for a physiologically relevant interaction between Bruce and cleaved Dcp-1. Based on this evidence, we decided to highlight Bruce in the manuscript, as it is the only factor for which both physiological and functional interactions with Dcp-1 are supported by multiple independent lines of evidence.

**Reviewer #3 (Public review):**

Summary:

The present paper by Shinoda et al. from the Miura group builds upon findings reported in an earlier study by the same team (Shinoda et al., PNAS, 2019), which identified a nonapoptotic role for the *Drosophila* executioner caspase Dcp-1 in promoting wing tissue growth. That earlier work attributed this function primarily to Dcp-1 and to Decay, a caspase structurally related to executioner caspases, but not to DrICE, the principal apoptotic executioner caspase. The authors further proposed that this non-apoptotic caspase activity operates independently of the initiator caspase Dronc.

In the current study, the authors both corroborate aspects of their previous findings and extend the investigation to mechanisms regulating Dcp-1 in this context. They identify roles for the giant IAP Bruce, two BCL-2 family members, and autophagy-related components in modulating nonapoptotic Dcp-1 activity. Moreover, they show that Bruce binds to a BIR-like peptide exposed upon Dcp-1 cleavage, but not to DrICE. The study further suggests that low levels of Dcp-1 activity promote wing tissue growth, whereas excessive activity induces cell death, as evidenced by impaired wing development following Dcp-1 overexpression. Overall, the manuscript provides several intriguing insights into the non-apoptotic regulation of the comparatively weak apoptotic executioner caspase Dcp-1 and complements the group's earlier work. However, several concerns remain regarding certain interpretations of the data and the experimental rigour of some of the results.

Strengths:

A major strength of the work is its systematic genetic and biochemical approaches, which combine tissue-specific manipulation with protein interaction mapping to explore how Dcp-1 is regulated. The identification of several regulatory factors, including an inhibitor of cell death protein and components linked to autophagy, provides a coherent framework for understanding how Dcp-1 activity might be tuned.

Weaknesses:

The evidence supporting some key claims remains incomplete. In particular, the type of cell death form induced when Dcp-1 is overexpressed is not clearly established, and additional tests would be needed to distinguish between the different cell death types.

Likely impact:

The study contributes to a growing body of work showing that proteins traditionally associated with cell death can have broader roles in tissue development. This conceptual advance is likely to be of interest to researchers studying growth control and tissue maintenance.

We sincerely thank the reviewer for their thoughtful and constructive evaluation of our study. In response to these concerns, we have performed additional experiments to clarify the nature of the cell death induced by *Dcp-1* overexpression. Based on the detection of cleaved Dcp-1, the detection of executioner caspase activity, and TUNEL assay, we now conclude that excessive Dcp-1 expression induces typical executioner caspase activity-dependent apoptotic cell death. Detailed explanations and experimental results are provided in the point-by-point responses below. Overall, we believe that these additions strengthen the manuscript by clarifying the dual roles of Dcp-1 in promoting tissue growth at low activity levels while triggering apoptosis when excessively activated.

Specific points:

(1) Nature of the wing ablation phenotype

A central concern is whether the wing ablation phenotype observed upon Dcp-1 overexpression truly reflects apoptotic cell death. The authors show in Figure 1c that nuclei in cells overexpressing Dcp-1, but not DrICE, zymogens are highly condensed, which is suggestive of apoptosis. However, it is equally plausible that this phenotype reflects a form of non-apoptotic, Dcp-1-dependent cell death (e.g. autophagy-dependent cell death). This distinction could be readily addressed using TUNEL labelling and direct caspase activity assays. The latter would be particularly informative, as it remains unclear whether zymogen Dcp-1 is capable of cleaving standard effector caspase reporters in vivo. Does the anti-cleaved Dcp-1 antibody detect Dcp-1 activation following overexpression of the Dcp-1 zymogen?

We thank the reviewer for this important point regarding the nature of cell death. We agree that nuclear condensation alone is not sufficient to conclude apoptotic cell death, and we therefore performed additional experiments. First, we performed TUNEL staining and detected robust TUNEL-positive signals in wing imaginal discs upon *Dcp-1* overexpression (new Figure 1D), supporting apoptotic DNA fragmentation. Second, to directly test whether *Dcp-1* overexpression leads to executioner caspase activity in vivo, we used two independent executioner caspase activity probes, GC3Ai (Schott et al., 2017; Zhang et al., 2013) and CD8::PARP::VENUS (Williams et al., 2006). Both probes showed clear executioner caspase activity-positive signals in wing imaginal discs upon *Dcp-1* overexpression (new Figure 1 – figure supplement 1C–F), demonstrating that *Dcp-1* overexpression leads to executioner caspase activity capable of cleaving standard substrates in vivo. In addition, staining with an anti-cleaved Dcp-1 antibody was positive upon Dcp-1 zymogen overexpression (new Figure 1 – figure supplement 1B), indicating that the overexpressed Dcp-1 zymogen is converted into its active form. Consistent with this result, western blot analysis revealed that Dcp-1 zymogen overexpression results in the appearance of a cleaved Dcp-1 (new Figure 1 – figure supplement 1A). Importantly, consistent with our original observation that the wing ablation phenotype is suppressed by expression of the caspase inhibitor *p35*, we further showed that *p35* overexpression completely abolished the appearance of cleaved Dcp-1 in western blot (new Figure 1 – figure supplement 1A), suggesting that Dcp-1 activation is mediated by self-cleavage. Taken together, these new results demonstrate that Dcp-1 zymogen overexpression induces typical executioner caspase activity-dependent apoptotic cell death. We have clarified this point in the revised manuscript and included the corresponding data.

(2) Role of Decay

In their earlier study, the authors identified Decay as another caspase influencing wing growth, albeit more modestly than Dcp-1. It is therefore unclear why this line of investigation was not pursued further in the current work. This omission is notable, as Decay is not implicated in apoptosis and, to date, no substantial physiological function has been assigned to this caspase in any system. At a minimum, this point should be discussed explicitly.

We thank the reviewer for the comment regarding the role of Decay. In our previous study (Shinoda et al., 2019), we demonstrated that both Dcp-1 and Decay promote wing tissue growth in a non-lethal manner. In the present study, however, we focused our analysis on Dcp-1. This decision was based on both technical and biological considerations. From a technical perspective, we had established TurboID knock-in lines and UAS overexpression lines for Dcp-1, Drice, and Dronc, whereas corresponding genetic tools are not available for Decay. From a biological standpoint, Dcp-1 exerts a stronger effect on wing growth than Decay, as shown in our previous work, and exhibits a dual functional spectrum: Dcp-1 promotes tissue growth at low activity levels, whereas excessive activation induces overt cell death. By contrast, Decay has not been implicated in cell death in wing imaginal discs (Kondo et al., 2006). Given that a central aim of the present study was to dissect how executioner caspase activity is differentially regulated to support nonlethal functions versus apoptotic cell death, we therefore focused on the two executioner caspases that are known to participate in apoptosis, Dcp-1 and Drice. We agree with the reviewer that Decay remains an intriguing caspase with largely unexplored physiological roles, and further investigation into its regulation and function will be an important direction for future studies. Importantly, Decay has been shown to mediate Hid-induced cell death in the DIAP1- and apoptosome-independent manner in differentiating photoreceptors and accessory cells of the eye (Leulier et al., 2006). In addition, although not required for cell death, Decay accounts for most of the caspase activity during metamorphic midgut programmed cell death, which is executed by autophagy (Denton et al., 2009). Thus, similar to Dcp-1, Decay might be an executioner caspase that can be regulated independently of the canonical apoptosome-mediated pathway, potentially involving autophagy-Bruce axis, and thereby contributing to the regulation of tissue growth. We have now discussed this point in the revised manuscript.

(3) Figure 2: Proximity labelling analysis

The authors use TurboID-mediated proximity labelling to reveal distinct Dcp-1- and DrICEassociated proteomes across tissues, with a particular focus on the wing disc. They further demonstrate that RNAi-mediated knockdown of the Dcp-1-associated proteins Sirt1 and Fkbp59 suppresses the wing ablation phenotype induced by Dcp-1 overexpression, suggesting that these factors are required for Dcp-1 activity. However, it should be clarified whether Bruce was identified as a Dcp-1 interactor in the proximity labelling dataset, given its proposed central regulatory role. In addition, further discussion of Fkbp59, its known functions and how it might mechanistically influence Dcp-1 activity would be valuable.

We thank the reviewer for the comment regarding the TurboID-based proximity labeling analysis and the interpretation of the identified Dcp-1-associated factors. With respect to Bruce, we clarify that Bruce was not identified as a Dcp-1 interactor in the TurboID proximity labeling dataset. Our co-immunoprecipitation analyses in S2 cells indicate that Bruce interacts specifically with cleaved Dcp-1, but not with the full-length, inactive form. In the revised manuscript, we confirmed by western blot that the majority of endogenously expressed Dcp-1 in wing imaginal discs exists in the full-length pro-form (new Figure 2 – figure supplement 2A). Therefore, the failure to detect Bruce in the TurboID experiment using full-length Dcp-1 as bait is expected, as this approach primarily labels proteins proximal to the inactive form of Dcp-1. To examine the Bruce/Dcp-1 interaction under more physiological conditions, we performed additional in vivo experiments. Using the mStayGold::V5-tag knock-in allele of *Bruce*, we overexpressed Dcp1::VENUS using *WP-Gal4* driver to induce Dcp-1 activation and assessed whether endogenously expressed full-length Bruce associates with Dcp-1 in wing imaginal discs. Following immunoprecipitation with anti-V5 antibody-conjugated magnetic agarose, we found that cleaved Dcp-1 signal was enriched by co-immunoprecipitation (new Figure 4I, J). These new data demonstrate that Bruce associates selectively with the cleaved, active form of Dcp-1 in vivo, thereby supporting the physiological relevance of the Bruce/Dcp-1 interaction. We have clarified this point in the revised manuscript and included the corresponding data.

FK506-binding proteins (FKBPs) are a conserved group of proteins known to bind FK506, an immunosuppressive drug. FKBPs contain FK domains, which correspond to peptidyl cis-trans isomerase (PPIase) domains. *Drosophila* Fkbp59 is an orthologue of the mammalian FKBP4 and FKBP5, both of which possess a C-terminal tetratricopeptide repeat (TPR) domain that functions independently of the PPIase domain by mediating protein-protein interactions. The mammalian orthologues of *Drosophila* Fkbp59 function as Hsp90 co-chaperones (GharteyKwansah et al., 2018). Importantly, loss of *Fkbp59* results in pupal lethality (Iki et al., 2020), which precludes further mechanistic analysis on Dcp-1 activation using adult wing phenotypes. To date, the involvement of Fkbp59 in caspase regulation has not been reported. Given that Fkbp59 functions as a co-chaperone, it may facilitate Dcp-1 activation by promoting proper folding, stability, or subcellular positioning of Dcp-1 or its regulatory factors. Importantly, Dcp1 proximal proteins are enriched in chaperone-related factors, including CCT2, CCT8, Droj2, CG16817, Fkbp59, Sgt1, and nudC; seven out of sixteen identified proximal proteins are chaperone-related. These observations suggest that Dcp-1 activity may be regulated by chaperone proteins or that Dcp-1 activity may be spatially restricted to regions enriched in chaperone machinery. Further analysis of the relationship between Dcp-1 activity and chaperone-related proteins will be important to elucidate the mechanisms and functions underlying non-lethal Dcp1 activation.

(4) Figure 3: Autophagy-related factors

Given that Sirt1 is known to promote autophagy, the authors next examine autophagy-related proteins and identify roles for Atg2, Atg8a, Debcl, and Buffy in Dcp-1 activation. Notably, these proteins do not promote cell death in the Hid-induced canonical apoptotic pathway. However, it is important to determine whether knockdown of Debcl, Buffy, Atg2, or Atg8a alone affects wing development in the absence of Dcp-1 overexpression, to exclude the possibility that these perturbations independently impair wing formation.

We thank the reviewer for the comment. To address whether knockdown of *Debcl*, *Buffy*, *Atg2*, or *Atg8a* independently affects wing development, we performed RNAi-mediated knockdown of each gene using the *WP-Gal4* driver in the absence of *Dcp-1* overexpression. Under these conditions, knockdown of *Debcl*, *Buffy*, *Atg2*, or *Atg8a* did not cause any detectable defects in wing morphology (new Figure 3 – figure supplement 1A), indicating that these autophagy-related factors specifically function to suppress Dcp-1-mediated cell death. We have clarified this point in the revised manuscript and included the corresponding data.

(5) Evidence for canonical autophagy

The involvement of autophagy would be more convincingly demonstrated by testing additional core autophagy genes, such as Atg7, Atg5, and Atg12, as well as performing a combined knockdown of Atg8a and Atg8b. Moreover, direct assessment of autophagy at the cellular level using established genetic reporters would substantially strengthen the conclusions.

We thank the reviewer for the constructive comment regarding the involvement of canonical autophagy. To further strengthen the evidence that autophagy is required for Dcp-1 activation, we examined additional core autophagy-related genes that function at distinct steps of the autophagy process, in addition to the previously tested *Atg2*, which mediates autophagosomal membrane expansion, and *Atg8a*, a core component directly associated with autophagosomal membranes. Specifically, we performed knockdown of genes including *FIP200/Atg17*, which is required for the initiation of autophagosome formation; *Atg9*, which is required for autophagosomal membrane nucleation; *Atg5*, which is required for autophagosomal membrane expansion through Atg12-Atg5-Atg16 ubiquitin-like conjugation system; and *Stx17*, which is required for autophagosome-lysosome fusion (Umargamwala et al., 2024). Because Atg8b is known to be specifically expressed in the male germline and is dispensable for autophagy, at least in fat body cells (Jipa et al., 2021), we did not further examine Atg8b in wing imaginal discs. Using *WPGal4* driver, knockdown of each of these genes significantly suppressed Dcp-1-induced wing ablation phenotype (new Figure 3 – figure supplement 1C), supporting a requirement for canonical autophagy components across multiple stages of autophagosome biogenesis in Dcp-1 activation. Importantly, knockdown of these autophagy-related genes alone did not affect wing morphology in the absence of *Dcp-1* overexpression (new Figure 3 – figure supplement 1B), as observed previously for Atg2 and Atg8a, suggesting the suppressive effects are specific to *Dcp-1* overexpression-dependent cell death. Together, these results indicate that inhibition of autophagy at any of several key steps can suppress Dcp-1-dependent cell death, demonstrating that intact canonical autophagy is required for Dcp-1 activation. In addition, to directly assess autophagy at the cellular level, we monitored autophagosome formation using mCherry::Atg8a reporter. Upon overexpression of Dcp-1::VENUS in the wing pouch region, we observed a clear accumulation of Atg8a-positive puncta in wing imaginal discs (new Figure 3C), demonstrating that *Dcp-1* overexpression induces autophagy in vivo. Together, these results provide both genetic and cellular evidence that canonical autophagy is activated upon *Dcp-1* overexpression and is required for Dcp-1-dependent cell death. We have clarified this point in the revised manuscript and included the corresponding data.

(6) Figures 4-5: Functional consequences

It would be informative to determine whether Synr, Debcl, or Buffy influence wing size on their own and whether their overexpression enhances wing growth.

We thank the reviewer for the suggestion regarding the functional consequences of Synr, Debcl, and Buffy on wing size. As requested, we knocked down *Debcl* or *Buffy* using *WP-Gal4* driver and found that this led to reduced wing size (new Figure 5 – figure supplement 1A), indicating that endogenous Debcl and Buffy promote wing growth potentially through regulating endogenous Dcp-1 activity. We have included the corresponding data in the revised manuscript. Because *Synr* RNAi did not show any detectable effect on the *Dcp-1* overexpression-induced phenotype (Figure 3A, B), we did not further examine the effect of *Synr* knockdown on wing development alone. Overexpression of *Synr* was not examined in this study. However, *Synr* overexpression has previously been reported to induce cell death in wing imaginal discs, resulting in malformed adult wings (Ikegawa et al., 2023), suggesting that increased Synr expression is likely to have deleterious rather than growth-promoting effects. Because Debcl and Buffy are both required for Synr-induced cell death, overexpression of *Debcl* or *Buffy* may lead to similar phenotypes. Therefore, we did not test *Debcl* or *Buffy* overexpression in the wing imaginal discs.

(7) Terminology and interpretation of cell death

Taken together, the results suggest that Dcp-1 zymogen overexpression induces a form of nonapoptotic cell death, potentially autophagy-dependent or related. The reviewer does not understand the authors' insistence on referring to this process as apoptosis. The authors should be more cautious in their terminology: there is no canonical versus non-canonical apoptosis; there is simply apoptosis. Without stronger evidence, these effects should not be described as apoptotic cell death.

We thank the reviewer for the important comment on terminology and interpretation of the cell death phenotype. As explained in our response to comment #1, we have performed additional experiments to clarify the nature of the cell death induced by *Dcp-1* overexpression. Based on the detection of cleaved Dcp-1, the detection of executioner caspase activity, and TUNEL assay, we now conclude that excessive Dcp-1 expression induces typical executioner caspase activity-dependent apoptotic cell death. At the same time, as explained in our response to comment #5, we provide both genetic and cellular evidence that canonical autophagy is activated upon *Dcp-1* overexpression and promotes Dcp-1 activation. We recognized that the phrase “Dcp1 activity-regulating alternative apoptosis signaling pathway” used in Figure 5L could be misleading, as it may imply the existence of an “alternative apoptosis”. To avoid this confusion, we have revised the figure legend to read “autophagy-facilitated alternative caspase activation pathway.”

**Reviewer #3 (Recommendations for the authors):**

Figure 1c should be annotated more clearly so that it is evident that the images shown are grouped by genotype.

We thank the reviewer for the helpful suggestion. We have added lines to Figure 1C to improve clarity by indicating that the images are grouped by genotype.

References

Choutka C, DeVorkin L, Go NE, Hou Y-CC, Moradian A, Morin GB, Gorski SM. 2017. Hsp83 loss suppresses proteasomal activity resulting in an upregulation of caspase-dependent compensatory autophagy. Autophagy 13:1573–1589.

Denton D, Shravage B, Simin R, Mills K, Berry DL, Baehrecke EH, Kumar S. 2009. Autophagy, not apoptosis, is essential for midgut cell death in *Drosophila*. Curr Biol 19:1741–1746.

DeVorkin L, Go NE, Hou Y-CC, Moradian A, Morin GB, Gorski SM. 2014. The *Drosophila* effector caspase Dcp-1 regulates mitochondrial dynamics and autophagic flux via SesB. J Cell Biol 205:477–492.

Domingues C, Ryoo HD. 2012. *Drosophila* BRUCE inhibits apoptosis through non-lysine ubiquitination of the IAP-antagonist REAPER. Cell Death Differ 19:470–477.

Ghartey-Kwansah G, Li Z, Feng R, Wang L, Zhou X, Chen FZ, Xu MM, Jones O, Mu Y, Chen S, Bryant J, Isaacs WB, Ma J, Xu X. 2018. Comparative analysis of FKBP family protein: evaluation, structure, and function in mammals and *Drosophila melanogaster*. BMC Dev Biol 18:7.

Ikegawa Y, Combet C, Groussin M, Navratil V, Safar-Remali S, Shiota T, Aouacheria A, Yoo SK. 2023. Evidence for existence of an apoptosis-inducing BH3-only protein, sayonara, in *Drosophila*. EMBO J 42:e110454.

Iki T, Takami M, Kai T. 2020. Modulation of Ago2 loading by Cyclophilin 40 endows a unique repertoire of functional miRNAs during sperm maturation in *Drosophila*. Cell Rep 33:108380.

Jipa A, Vedelek V, Merényi Z, Ürmösi A, Takáts S, Kovács AL, Horváth GV, Sinka R, Juhász G. 2021. Analysis of *Drosophila* Atg8 proteins reveals multiple lipidation-independent roles. Autophagy 17:2565–2575.

Kondo S, Senoo-Matsuda N, Hiromi Y, Miura M. 2006. DRONC coordinates cell death and compensatory proliferation. Mol Cell Biol 26:7258–7268.

Kornbluth S, White K. 2005. Apoptosis in *Drosophila*: neither fish nor fowl (nor man, nor worm). J Cell Sci 118:1779–1787.

Leulier F, Ribeiro PS, Palmer E, Tenev T, Takahashi K, Robertson D, Zachariou A, Pichaud F, Ueda R, Meier P. 2006. Systematic in vivo RNAi analysis of putative components of the *Drosophila* cell death machinery. Cell Death Differ 13:1663–1674.

Ryoo HD, Baehrecke EH. 2010. Distinct death mechanisms in *Drosophila* development. Curr Opin Cell Biol 22:889–895.

Schott S, Ambrosini A, Barbaste A, Benassayag C, Gracia M, Proag A, Rayer M, Monier B, Suzanne M. 2017. A fluorescent toolkit for spatiotemporal tracking of apoptotic cells in living *Drosophila* tissues. Development 144:3840–3846.

Shinoda N, Hanawa N, Chihara T, Koto A, Miura M. 2019. Dronc-independent basal executioner caspase activity sustains *Drosophila* imaginal tissue growth. Proc Natl Acad Sci U S A 116:20539–20544.

Tenev T, Zachariou A, Wilson R, Ditzel M, Meier P. 2005. IAPs are functionally non-equivalent and regulate effector caspases through distinct mechanisms. Nat Cell Biol 7:70–77.

Umargamwala R, Manning J, Dorstyn L, Denton D, Kumar S. 2024. Understanding developmental cell death using *Drosophila* as a model system. Cells 13:347.

Vernooy SY, Chow V, Su J, Verbrugghe K, Yang J, Cole S, Olson MR, Hay BA. 2002. *Drosophila* Bruce can potently suppress Rpr- and Grim-dependent but not Hid-dependent cell death. Curr Biol 12:1164–1168.

Williams DW, Kondo S, Krzyzanowska A, Hiromi Y, Truman JW. 2006. Local caspase activity directs engulfment of dendrites during pruning. Nat Neurosci 9:1234–1236.

Zhang J, Wang X, Cui W, Wang W, Zhang H, Liu L, Zhang Z, Li Z, Ying G, Zhang N, Li B. 2013. Visualization of caspase-3-like activity in cells using a genetically encoded fluorescent biosensor activated by protein cleavage. Nat Commun 4:2157.
